# Recurrent prolapse of toric implantable collamer lens after blunt ocular trauma under mesopic conditions

**DOI:** 10.1002/ccr3.2055

**Published:** 2019-02-17

**Authors:** Yuki Takagi, Tomoaki Nakamura, Kazuo Ichikawa, Takashi Kojima

**Affiliations:** ^1^ Department of Ophthalmology Iida Municipal Hospital Nagano Japan; ^2^ Nagoya Eye Clinic Nagoya Japan; ^3^ Chukyo Eye Clinic Nagoya Japan; ^4^ Department of Ophthalmology Keio University School of Medicine Tokyo Japan

## Abstract

Implantable collamer lenses (ICL) carry a risk of prolapse caused by direct ocular trauma, especially in mesopic conditions and when oversized ICLs are implanted. We recommend early surgical repositioning, as well as patient education that encourages goggle use during active sports.

## 1 INTRODUCTION

This is a unique report of recurrent prolapse of an implantable collamer lens (ICL) caused by ocular trauma under mesopic conditions in a patient with a high vault distance. Early repositioning surgery was performed safely and without subsequent complications. Thus, such surgery may be necessary to avoid vision‐threatening complications.

The ICL is a posterior chamber phakic intraocular lens that can safely and effectively treat myopia of all severities.^1^, ^2^, ^3^, ^4^ The number of patients undergoing ICL implantation increases annually, and 785 000 eyes had received implantation by 2017 (STAAR Surgical corporate material). The earlier V4 ICL model was associated with several complications, including intraocular pressure elevation (due to pupillary block) and cataract formation. However, complications rarely occur after implantation of the more current V4c and V5 models.^1^, ^3^, ^4^

Ocular trauma can affect visual function after laser refractive surgery.^5^, ^6^ However, few reports have described the effect of blunt ocular trauma on eyes with ICLs. The present case is particularly unique because it shows that an ICL can be repeatedly prolapsed by ocular trauma during mesopic conditions. Moreover, it suggests that early surgical intervention should be performed to successfully manage ICL haptic prolapse after blunt ocular trauma.

## 2 CASE REPORT

**Figure 1:**
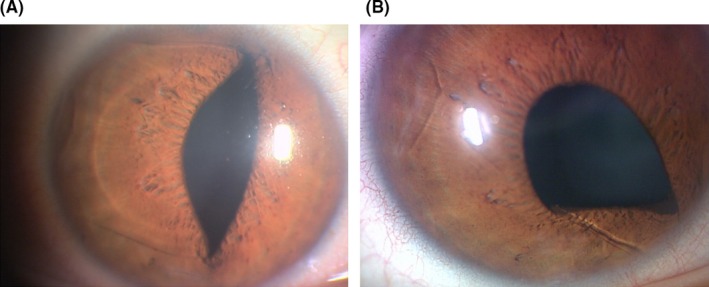
Slit‐lamp microscopy photograph of an eye that incurred blunt trauma during a futsal game, resulting in prolapse of a toric implantable collamer lens (TICL). A, Eleven months after the initial implantation, blunt trauma to the right eye resulted in TICL prolapse. The superotemporal and inferotemporal haptics had prolapsed into the anterior chamber. B, The patient was hit in the right eye again 26 mo after initial TICL implantation. The inferonasal haptic had prolapsed into the anterior chamber

A 39‐year‐old man presented to our outpatient clinic to undergo ICL implantation surgery. He had no history of systemic or ocular diseases other than refractive error. His preoperative uncorrected visual acuity (UCVA) was 20/500 in each eye, while his distance‐corrected visual acuity (DCVA) was 20/13 (S, −7.00; Cyl, −1.75 × 20°) in the right eye and 20/13 (S, −6.25; Cyl, −2.5 × 10°) in the left eye. The intraocular pressure (IOP) was 16 and 15 mm Hg, and the corneal endothelial cell density was 3118 and 3318 cells/mm^2^ in the patient's right and left eyes, respectively. In the right and left eye, respectively, the pupil sizes were 8 and 8 mm in a dark examination room, and 5.7 and 5.8 mm in a bright examination room; the anterior chamber depths (from the corneal endothelium to the anterior lens capsule) were 3.58 and 3.55 mm, the white‐to‐white (WTW) diameters were 11.4 and 11.4 mm, the sulcus‐to‐sulcus (STS) distances were 11.7 and 11.68 mm, and the corneal thicknesses was 539 and 528 μm. Two months before the toric ICL (TICL) implantation surgery, laser iridotomy was performed in both eyes. Based on the calculations of the TICL software (STAAR Surgical Company, Monrovia, CA, USA), we chose the TICMV4 model, with a power of −13.5 + 3.5 × 89° in the right eye and −13.5 + 2.5 × 101° in the left eye, and a diameter of 12.6 mm in both eyes. The TICLs were implanted without any complications at 16° and 0° anticlockwise from the horizontal meridian in the right and left eyes, respectively. In both eyes, the 3‐month postoperative UCVA was 20/10; the DCVA was also 20/10 (S, +1.00; Cyl, −0.50 × 135° in the right eye and S, +0.50; Cyl. −0.50 × 20° in the left eye). The IOP was 12 and 13 mm Hg, respectively, and the corneal endothelial cell density 3 months after surgery was 3125 and 3086 cells/mm^2^. The central vault, which is the distance between the TICL and crystalline lens, was 0.9 and 1.05 mm 3 months after surgery. Although the central vault was high, we followed it without surgical interventions because the angle was open and there were no complications such as IOP rise, iris contact with the corneal endothelium, or pigment dispersion syndrome. Eleven months after the initial ICL implantation surgery, the patient developed blurred vision in his right eye after being struck by another player's hand during a futsal match. The patient presented to the outpatient clinic the day after the injury. The UCVA in his right eye was 20/13, while the DCVA was 20/13 (S, +1.25; Cyl, −0.50 × 130°). Slit‐lamp examination revealed mild inflammation in the anterior chamber, and both the superior and inferior temporal haptics of the ICL were dislodged into the anterior chamber and entrapped within the pupil, without touching the corneal endothelium (Figure 1A). We performed repositioning surgery on the same day. After filling the anterior chamber with viscoelastic material, we used the ICL manipulator from the preexisting wound to reposition the ICL behind the iris. The patient used 1.5% levofloxacin, 0.1% bromfenac sodium hydrate, and 0.1% betamethasone ophthalmic solutions for 1 week after the operation. One month after the repositioning surgery, his UCVA was 20/13 and his DCVA was 20/13 (S, +0.75; Cyl, −0.75 × 135°). The blurred vision in his right eye was resolved. The IOP in the affected eye was 13 mm Hg; the corneal endothelial cell density was 3001 cells/mm^2^, and the vault distance was 0.82 mm. No complications such as iris damage, cataract formation, or ICL rotation were noted. Two years and 2 months after the initial TICL implantation surgery, the patient again developed blurred vision in his right eye after being struck by another player's foot during a futsal match. The UCVA in his right eye was 20/17, and the DCVA was 20/17 (S, +0.75; Cyl, −0.75 × 135°). We observed mild inflammation in the anterior chamber, and the inferonasal haptic of the ICL was dislodged into the anterior chamber and entrapped within the pupil, without touching the corneal endothelium (Figure 1B). We performed repositioning surgery the next day. The patient used 1.5% levofloxacin, 0.1% bromfenac sodium hydrate, and 0.1% fluorometholone ophthalmic solutions for 1 month after the operation. One month after surgery, his UCVA was 20/10, while his DCVA was 20/10 (S, +0.50; Cyl, −0.50 × 105°). The IOP was 17 mm Hg, the corneal endothelial cell density was 3050 cells/mm^2^, and the vault distance was 0.79 mm. No complications associated with the repositioning surgery or trauma were observed. We observed pigment deposition on the posterior surface of the ICL after the surgery, but the amount of the pigment had not changed 1 year after injury. We explained to the patient the risk of ICL prolapse during futsal at night, advising him to use protective glasses.

## 3 DISCUSSION

Several cases of ICL prolapse have been reported in the literature.^7^, ^8^, ^9^, ^10^, ^11^ Most such cases have had good visual outcomes with no complications following ICL repositioning surgery,^7^, ^8^, ^9^, ^11^ similar to the present case. However, Espinosa et al^10^ reported severe corneal endothelial cell damage. In their study, the ICL prolapsed into the anterior chamber, was entrapped by the pupil, and contacted the corneal endothelium. Three months after repositioning surgery, bullous keratopathy developed, and the patient ultimately underwent Descemet stripping automated endothelial keratoplasty (DSAEK). We suspect that ICL contact with the corneal endothelium results in endothelial damage, ultimately leading to the bullous keratopathy. Additionally, Espinosa et al performed repositioning surgery 3 days after ICL dislocation, indicating that early repositioning surgery is necessary.

The current patient developed ICL prolapse following blunt trauma to the eye during a nighttime futsal match. The game was played in an indoor futsal stadium equipped with several lights; field luminance fell within the mesopic range. Given that the pupils generally dilate when the sympathetic system is activated, as when playing sports, we suspect that our patient had enlarged pupils at the time of trauma. This allowed the ICL to pass into the anterior chamber via the pupil. Three previous case reports^8^, ^9^, ^10^ have described patients with ICL prolapse following an injury incurred under mesopic conditions. These reports did not document the details of the situation regarding mesopic pupil size. However, a large pupil may have contributed to ICL prolapse. Further investigations are needed to determine whether miotic agents can prevent ICL dislocation, particularly under dim lighting conditions when the pupil is dilated.

Previous reports^7^, ^8^, ^9^, ^10^, ^11^ have documented post‐traumatic ICL dislocations 4 months to 6 years after the initial ICL implantation, and the patient in the current case had ICL dislocations 11 and 26 months after initial ICL implantation. None of these cases showed iris damage after ICL dislocation and repositioning, so we concluded that adhesions do not form between the ICL and the iris or ciliary sulcus tissues, even a long time after ICL implantation, because the ICL has high biocompatibility. Indeed, it may have been this high biocompatibility that ultimately contributed to ICL prolapse.

In previous reported cases, as in the present case, ICL prolapse was caused by severe blunt ocular trauma—a punch,^9^ unspecified blunt ocular trauma,^7^, ^10^ and a strike with a pipe.^11^ Thus, strong direct impact to the eye may change the shape of the eyeball, leading to ICL prolapse. However, Kong et al^8^ reported a case in which severe occipital trauma, which only indirectly impacts the eye, caused ICL prolapse. Therefore, further investigation is needed to better understand the mechanisms by which ICL prolapse occurs.

Additionally, in the current case, the vault distance (between the ICL and the crystalline lens) was high before trauma occurred. No prior reports have associated high vault distance with ICL dislocation after blunt trauma. Nonetheless, it may have played a role in the current case. When an oversized ICL is implanted, a traumatic impact may have a greater effect than when a proper‐sized ICL is implanted. Furthermore, eyes with a high vault distance may be more susceptible to corneal endothelial damage after trauma, because the ICL is located closer to the corneal endothelium than in eyes with a lower vault distance. Further studies are needed to evaluate the effect of vault distance on the incidence of ICL prolapse and corneal endothelial complications.

In the present case, both UCVA and DCVA were slightly decreased following ICL prolapse. However, even after two ICL repositioning procedures, both values returned to pretrauma levels, suggesting that a tilted ICL optic (as during ICL prolapse) increases higher‐order aberrations and causes temporary decreases in visual acuity.

McCauley et al^12^ reported a case in which an ICL remained in position after severe trauma (grenade explosion)—the patient was wearing protective glasses, which may prevent a direct impact to the eye and ICL prolapse.

The present case is important because it demonstrates that blunt trauma to the eye confers a risk for ICL prolapse into the anterior chamber, even if the ICL was implanted years ago. It also suggests that mesopic conditions, direct blunt ocular trauma, and high vault distance are risk factors of ICL prolapse. Furthermore, ICL prolapse can occur repeatedly, because the ICL does not form adhesions with the iris or ciliary sulcus tissues, because the ICL has high biocompatibility. After repositioning surgery, visual acuity was restored to preoperative levels, and no complications, such as endothelial loss, were observed, suggesting that early repositioning surgery is necessary. Additionally, wearing protective glasses can help prevent ICL prolapse, especially in patients who play sports with a risk of blunt trauma. We believe our report will be useful to refractive surgeons who implant ICLs, as well as to general ophthalmologists, who may encounter patients with ICL prolapse in an emergency setting.

## CONFLICT OF INTEREST

The authors have no conflict of interests to declare.

## AUTHOR CONTRIBUTIONS

YT, KI, and TK: involved in study conception and design. YT, TN, and TK: involved in acquisition of data. YT, TN, KI, and TK: involved in analysis and interpretation of data and final approval. YT and TK: involved in drafting or revising manuscript.
